# Matricellular proteins: Potential biomarkers in head and neck cancer

**DOI:** 10.1002/ccs3.12027

**Published:** 2024-04-09

**Authors:** Yunsheng Wang, Xudong Liu, Xingyue Wang, Jiyong Lu, Youxin Tian, Qinjiang Liu, Jincai Xue

**Affiliations:** ^1^ Department of Head and Neck Surgery Gansu Provincial Cancer Hospital Lanzhou China

**Keywords:** biomarkers, extracellular matrix, head and neck cancer, matricellular proteins

## Abstract

The extracellular matrix (ECM) is a complex network of diverse multidomain macromolecules, including collagen, proteoglycans, and fibronectin, that significantly contribute to the mechanical properties of tissues. Matricellular proteins (MCPs), as a family of non‐structural proteins, play a crucial role in regulating various ECM functions. They exert their biological effects by interacting with matrix proteins, cell surface receptors, cytokines, and proteases. These interactions govern essential cellular processes such as differentiation, proliferation, adhesion, migration as well as multiple signal transduction pathways. Consequently, MCPs are pivotal in maintaining tissue homeostasis while orchestrating intricate molecular mechanisms within the ECM framework. The expression level of MCPs in adult steady‐state tissues is significantly low; however, under pathological conditions such as inflammation and cancer, there is a substantial increase in their expression. In recent years, an increasing number of studies have focused on elucidating the role and significance of MCPs in the development and progression of head and neck cancer (HNC). During HNC progression, there is a remarkable upregulation in MCP expression. Through their distinctive structure and function, they actively promote tumor growth, invasion, epithelial‐mesenchymal transition, and lymphatic metastasis of HNC cells. Moreover, by binding to integrins and modulating various signaling pathways, they effectively execute their biological functions. Furthermore, MCPs also hold potential as prognostic indicators. Although the star proteins of various MCPs have been extensively investigated, there remains a plethora of MCP family members that necessitate further scrutiny. This article comprehensively examines the functionalities of each MCP and highlights the research advancements in the context of HNC, with an aim to identify novel biomarkers for HNC and propose promising avenues for future investigations.

## Graphical abstract

Schematic diagram of the role of matricellular proteins in head and neck cancer.

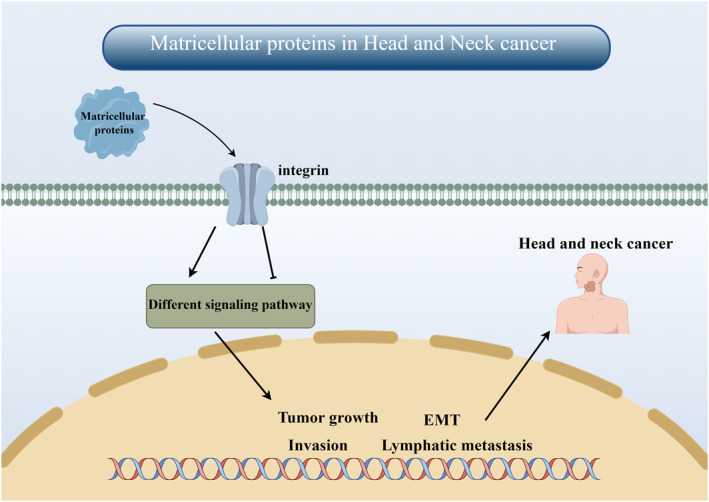

## 1 INTRODUCTION

Head and neck cancer (HNC) ranks as the sixth most prevalent malignancy worldwide.^1^, ^2^ HNC primarily originates from the mucosal lining of the upper respiratory tract, predominantly affecting tissues within the oral cavity, pharynx, and larynx.^1^, ^3^ This aggressive malignant tumor exhibits a substantial burden in terms of morbidity and mortality rates.^4^ Head and neck squamous cell carcinoma (HNSCC) represents over 90% of all HNC cases.^5^ The classical major risk factors associated with HNC encompass prolonged tobacco smoking and alcohol consumption habits.^6^ Furthermore, individuals afflicted with genetic disorders such as Li‐Fraumeni syndrome or Fanconi anemia exhibit heightened susceptibility to developing this form of cancer.^7^ However, over the past few decades, human papillomavirus (HPV) infection has emerged as a novel risk factor for HNC. HPV (+) HNC predominantly occurs in the oropharynx, where there is minimal or no exposure to tobacco.^8^ Notably, patients with HPV (+) HNC exhibit a more favorable prognosis and heightened sensitivity to radiotherapy and anticancer medications.^9^ The current standard treatment for most individuals with HNSCC involves surgical intervention. Postoperative concurrent chemoradiotherapy employing platinum‐based agents is widely acknowledged as the accepted standard of care for high‐risk patients at risk of recurrence.^10^, ^11^ However, it is important to acknowledge that therapies such as radiation and chemotherapy can induce toxicity in other organs, thereby compromising the overall quality of life. Consequently, there is a growing imperative to identify molecular biomarkers capable of predicting HNSCC progression for improved survival outcomes and the discovery of novel treatment targets. Matricellular proteins (MCPs) represent a family of nonstructural proteins that intricately regulate various extracellular matrix (ECM) functions.^12^ In contrast to the persistent presence of structural proteins within the ECM, MCP expression is tightly regulated to exert their potent biological functions.^13^ Recent studies have increasingly reported on the involvement of MCPs in numerous cancer diseases' occurrence and development,^14^ particularly in HNSCC. Therefore, this review aims to comprehensively examine each MCP's function and research advancements within the MCP family specifically pertaining to HNSCC, with an ultimate goal of identifying new biomarkers for HNC.

## 2 CAUSES AND TREATMENT OF HNC

HNC originates in the upper aerodigestive tract, encompassing the lips, oral cavity, salivary glands, larynx, nasopharynx, hypopharynx, and oropharynx. It stands as the sixth most prevalent cancer type worldwide and ranks seventh among leading causes of death.^7^ Annually, approximately 900,000 cases are diagnosed with this form of cancer; however, only around half of these patients survive beyond 5 years.^15^ Epidemiological studies have unveiled diverse risk factors for HNC classified by the International Agency for Research on Cancer under the World Health Organization. These factors include smoking habits, alcohol consumption patterns, exposure to environmental pollutants as well as infectious viral agents such as HPV and EB virus.^2^ According to reports, smoking has been associated with a 5–25 times increased incidence of HNC, with a clear correlation observed between the amount and duration of smoking.^16^, ^17^ Similarly, smokeless tobacco has been found to increase the incidence of HNC by 2–4 times.^18^ However, the impact of e‐cigarettes on HNC risk remains uncertain. Tobacco contains numerous carcinogens such as polycyclic aromatic hydrocarbons and nitrosamines. These carcinogens generate reactive metabolites that accumulate and form covalent DNA adducts, leading to DNA damage. Smoking also induces the production of inflammatory factors and chemokines in exposed tissues, which play a crucial role in promoting proliferation, angiogenesis, and ultimately carcinogenesis.^2^ Excessive alcohol consumption represents a significant risk factor for HNC. Alcohol intake independently doubles the incidence of HNC, while also exhibiting a synergistic effect with smoking.^19^ As a solvent for carcinogens, alcohol enhances the exposure of epithelial cells to these harmful substances. Furthermore, the metabolites of alcohol contribute to DNA damage by generating DNA adducts.^20^, ^21^

In addition to smoking and alcohol consumption, genetic factors independently contribute to the increased susceptibility of HNC. Fanconi anemia, a rare genetic disorder characterized by impaired DNA repair mechanisms, significantly heightens the risk of developing HNC in affected individuals; however, the precise underlying mechanism remains elusive.^22^

Evidence supporting HPV as a causative agent of HNC emerged during the 1990s.^23^ Numerous studies have reported that high‐risk subtypes, such as HPV‐16, 18, 31, and 33, exert oncogenic effects by integrating their viral DNA into host cells and expressing the E6 and E7 oncogenes.^24^ Oropharyngeal cancers are predominantly associated with HPV infection. The E6 protein induces early degradation of the tumor suppressor protein p53,^25^ while also preventing apoptosis through degradation of pro‐apoptotic proteins in a p53‐independent manner.^26^ Additionally, the E7 protein directly interacts with the tumor suppressor Rb, leading to release of the transcription factor E2F and subsequent transcription of DNA‐methyltransferase 1 (DNMT1). Overexpression of DNMT1 results in DNA hypermethylation, a prevalent phenomenon observed in HPV (+) oropharyngeal cancer.^27^ Both E6 and E7 have the ability to induce cell proliferation individually,^24^ and their interaction leads to the manifestation of pathogenic effects.

Current treatments for HNC encompass surgical interventions, radiation therapy, chemotherapy, or their combinations. Despite significant advancements in treatment modalities and outcomes, the long‐term survival rate of patients remains notably low.^4^, ^28^ Presently, only two molecular‐targeted therapies have gained approval for HNC treatment: cetuximab and monoclonal anti‐PD‐L1 agents.^7^ Consequently, it is imperative to identify molecular biomarkers capable of predicting the progression of HNSCC, thereby enhancing survival rates and discovering novel therapeutic targets.

## 3 MCP FAMILY

**FIGURE 1:**
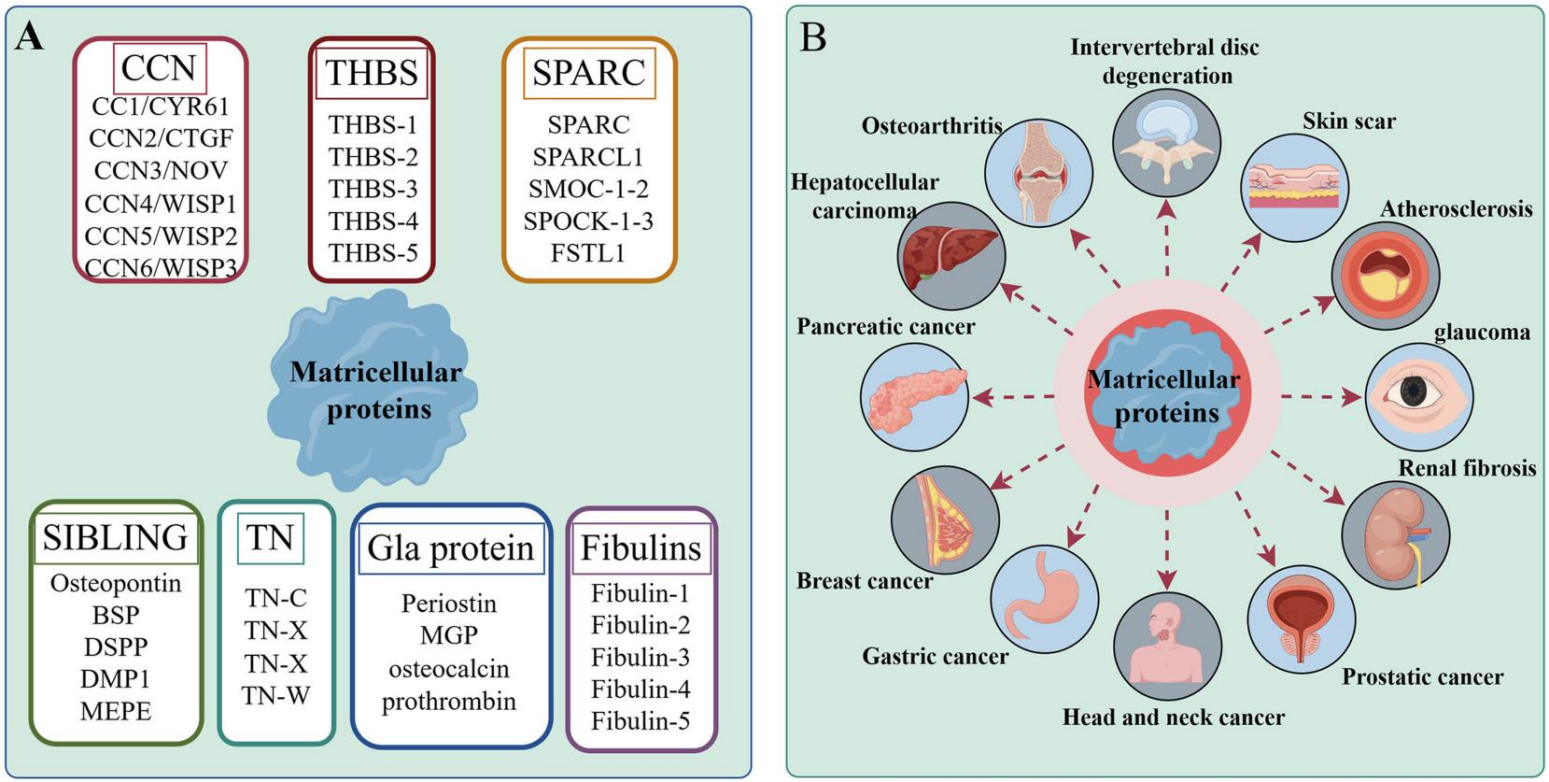
Classification of MCPs and human diseases involved. (A) Classification of MCPs. (B) Human diseases in which MCPs are involved. MCPs, matricellular proteins.

The concept of MCPs was initially introduced by Bornstein et al. during their investigation of two novel protein types, namely thrombospondins (THBS) and the secreted proteins acid and rich in cysteine family (SPARC). Subsequently, an increasing number of proteins with analogous functions were classified as MCPs.^29^, ^30^ Bornstein defined this group of proteins as a modular ensemble of extracellular proteins that exert their function through binding to matrix proteins, cell surface receptors, or other molecules such as cytokines and proteases, which subsequently engage in cellular surface interactions. MCPs encompass a collection of ECM‐associated proteins. The ECM occupies a critical niche in regulating diverse cellular processes, encompassing survival, proliferation, and migration. Consequently, it plays an indispensable role in fundamental physiological phenomena like cell development, tissue homeostasis, and tissue remodeling.^31^ While collagen, fibronectin, and laminin serve as classic ECM proteins with structural functions,^29^, ^32^ the dynamic nature of the ECM is governed by nonstructural matrix cellular proteins. In contrast to the persistent presence of structural proteins within the ECM framework, the expression of these nonstructural counterparts—known as MCPs—is meticulously regulated to precisely tailor their roles during tissue maintenance and repair processes.^33^ MCPs modulate diverse cellular functions through their interactions with cell surface receptors, proteases, hormones, and ECM.^12^ It is noteworthy that the expression levels of MCPs in adult steady‐state tissues are typically low; however, under pathological conditions such as inflammation or cancer, their expression significantly increases.^14^ Based on their analogous structures and functionalities, these MCPs can be categorized into seven families: THBS, fibulins, SPARC family, the centralized coordination network (CCN) family, tenascins (TNs), the small integrin‐binding ligand N‐linked glycoprotein (SIBLING) family, and the Gla protein family.^34^, ^35^ Nevertheless, recent studies have reported several emerging MCPs that remain unclassified. Figure 1A is the classification of MCPs protein family.

In recent years, there has been extensive research on the structure and function of MCPs. Numerous studies have increasingly highlighted the significant role of MCPs in the pathogenesis and progression of various diseases within the human body.^31^ Consequently, MCPs have emerged as valuable molecular markers for numerous diseases. Specifically, lumbar intervertebral disc degeneration has been confirmed to involve MCPs as crucial molecular targets.^36^ Notably, Periostin can activate the Wnt/β‐catenin signaling pathway to induce apoptosis in nucleus pulposus cells.^37^ Additionally, Periostin forms a positive feedback loop with the NF‐κB signaling pathway, thereby accelerating aging and apoptosis in nucleus pulposus cells and ultimately promoting intervertebral disc degeneration.^38^ CCN family proteins are recognized as molecular targets in osteoarthritis and rheumatoid arthritis, exerting their own regulatory functions to control chondrocyte degeneration.^39^, ^40^ Moreover, MCPs play a pivotal role not only in orthopedic diseases but also in various other conditions including skin scar regeneration,^41^ atherosclerosis,^12^ glioblastoma,^42^ and glaucoma.^43^ Furthermore, extensive evidence confirms the involvement of MCPs in cancer initiation and progression. In particular, diverse MCPs such as CCN family proteins, THBS, and SPARC have emerged as independent prognostic markers for unfavorable cancer outcomes.^44^, ^45^, ^46^ In addition, MCPs can also interact with integrins, cell surface receptors, proteases, etc., thereby modulating various signaling pathways involved in angiogenesis, cell migration and invasion, as well as epithelial‐to‐mesenchymal transition (EMT), which are crucial for cancer initiation and progression.^47^, ^48^ Recent studies have extensively explored the intricate association between MCPs and HNC. Therefore, this review aims to shed light on the current understanding of MCPs in HNC by elucidating their emerging roles.

## 4 CCN FAMILY

The CCN family proteins in MCPs encompass the homologous matrix protein family and consist primarily of six proteins, namely cysteine‐rich 61 (CYR61/CCN1), connective tissue growth factor (CTGF/CCN2), nephroblastoma‐overexpressed (NOV/CCN3), Wnt‐1 induced secreted protein‐1 (WISP1/CCN4), Wnt‐1 induced secreted protein‐2 (WISP2/CCN5) and Wnt‐1 induced secreted protein‐3 (WISP3/CCN6).^49^ Each CCN protein comprises four distinct functional domains, exhibiting significant homology and functional similarities. Despite their highly similar primary structures, the three‐dimensional structures of these CCN proteins exhibit substantial differences.^50^, ^51^ Due to their distinctive four‐module organizational structure, CCN family proteins possess remarkable biological properties that are either independent or complementary to each other.^52^ These proteins play crucial roles in angiogenesis, wound healing, inflammation, and have been initially characterized as regulators of cell proliferation, adhesion, migration, differentiation, and cytokines involved in ECM differentiation and production.^53^, ^54^ Although the involvement of the CCN protein family in HNC remains relatively unexplored in literature thus far, existing studies have unveiled emerging disease‐related functions for this protein family.

CCN2, also known as CTGF, is the most extensively studied and well‐known member of the CCN protein family. In a previous study conducted by Mullis et al.,^55^ CTGF expression in both HNSCC tissues and normal oral mucosa tissues was detected using real‐time quantitative PCR. The results revealed a significant increase in CTGF expression within HNCSS tissues, and subsequent immunohistochemical localization analysis demonstrated its presence in stromal fibroblasts, tumors, and vascular endothelial cells. These findings suggest that CTGF may play a crucial role in the initiation and progression of HNC. Another study by Kikuchi et al.^56^ further supported these observations through immunohistochemistry, revealing undetectable levels of CTGF immunoreactivity in non‐neoplastic head and neck squamous epithelium but higher levels observed in some neoplastic head and neck squamous epithelium samples. Furthermore, Kikuchi et al. demonstrated that CTGF was significantly associated with unfavorable prognosis in stage IV HNC patients, underscoring the prognostic significance of CTGF. A study investigating the co‐culture of head and neck squamous cells with mesenchymal stromal cells (MSCs) highlighted that MSCs predominantly secrete CTGF, while tumor cell‐derived TGF‐β acts as the primary regulator for enhanced CTGF production in MSCs.^57^ Additionally, Chang et al. reported that CTGF has the ability to induce MET in HNSCC and facilitate tumor growth.^58^ In addition, it was discovered that CTGF significantly augmented the stem cell characteristics of HNC cells and upregulated the expression of multiple pluripotency genes. Mechanistic investigations have revealed that CTGF induces c‐Jun expression via integrin αvβ3, which in turn directly activates the transcription of pluripotent genes NANOG, SOX2, and POU5F1. Furthermore, in samples obtained from HNC patients, a positive correlation was observed between CTGF expression levels and CDH1, NANOG, SOX2, as well as POU5F1. These compelling pieces of evidence strongly suggest that CCN2 may serve as a pivotal molecule in both the development and prognosis of HNC. Therefore, future research endeavors should prioritize elucidating the precise role played by CCN2 in HNC pathogenesis.

CCN4‐6, also known as WISP1‐3, are three target genes of the Wnt signaling pathway. While WISP1 plays a crucial role in development, mounting evidence suggests its involvement in carcinogenesis.^59^ In esophageal squamous cell carcinoma studies, WISP1 has been identified as an independent prognostic factor for poor overall survival and resistance to radiotherapy.^60^, ^61^ Recent research has linked WISP1 closely with HNSCC. Kempen et al.^62^ utilized multiplex ligation probe amplification to investigate the copy number status of 36 oncogenes and tumor suppressor genes in oropharyngeal and oral squamous cell carcinomas. Multivariate survival analysis revealed an upregulation of WISP1 expression in two types of squamous cell carcinomas. Furthermore, the study identified that increased WISP1 gain at 8q24.22 serves as a predictive factor for deteriorating disease‐free survival in squamous cell carcinoma patients. Moreover, multiple investigations employing RT‐PCR, immunohistochemistry, and lentiviral transfection have consistently confirmed the association between WISP1 and the occurrence and progression of advanced HNSCCs such as esophageal squamous cell carcinoma and oral squamous cell carcinoma. Notably, these studies have demonstrated a significant correlation with certain prognostic implications,^63^, ^64^, ^65^ wherein WISP1 primarily facilitates tumor cell invasion while inhibiting tumor cell apoptosis.^63^ Additionally, Wang^66^ and Song^67^ reported through Western Blot analysis, PCR assays, and lentivirus transfection experiments that WISP1 promotes HNSCC invasion and metastasis while modulating glycolysis and chemoresistance via YAP1/TEAD1/GLUT1 signaling pathway as well as TGF‐β‐Smad2/3 signaling pathway regulation. In addition to its role in regulating multiple signaling pathways involved in the occurrence and development of HNC, WISP1 has been shown to interact with non‐coding RNA, thereby influencing cancer progression. Yang et al. demonstrated that knockdown of long non‐coding RNA CCAT2 can effectively inhibit the activity of WISP1, leading to suppression of esophageal squamous cell carcinoma occurrence and metastasis.^68^ Furthermore, Wang et al.^69^ identified WISP1 as a direct target of miR‐384 through dual‐luciferase reporter experiments and observed up‐regulation of WISP1 upon inhibition of miR‐384. Subsequently, they discovered that miR‐384 directly targets WISP1 to modulate cell proliferation and apoptosis in HNSCC.

In addition to WISP1, the pivotal roles of WISP2 and WISP3 have been implicated in various types of human cancer diseases. Chai et al.^70^ manipulated the expression of WISP2 through lentiviral transfection, observing that its overexpression significantly impeded tumor growth in mouse models. Conversely, down‐regulation of WISP2 promoted esophageal squamous cell carcinoma cell growth while inhibiting apoptosis and facilitating cell migration and invasion. These effects were mediated by the targeting of ERK and E‐cadherin pathways by WISP2. Furthermore, emerging evidence suggests a potential tumor suppressor role for WISP3 in several cancers.^71^, ^72^ Yu et al.^73^ demonstrated that the overexpression of WISP3 not only impeded the proliferation and migration of esophageal squamous cell carcinoma cells in vitro but also effectively suppressed tumor growth and metastasis in vivo. Mechanistically, WISP3 exerted its biological function by inhibiting the IGF‐2‐IGF1R signaling cascade and attenuating AKT signaling activity.

**FIGURE 2:**
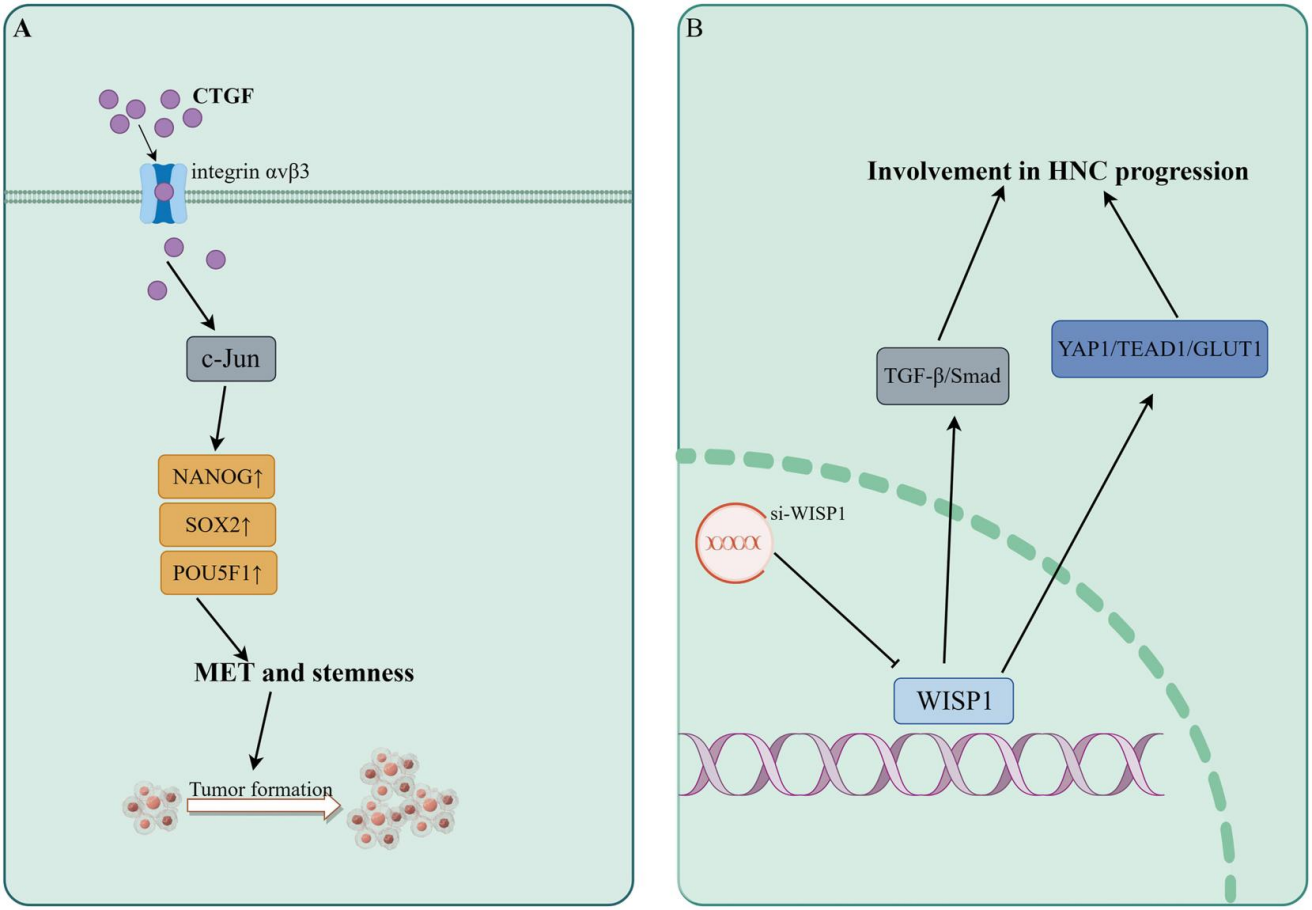
Mechanism of CCN protein family involved in HNC. (A) Mechanism of CTGF involvement in HNC. (B) Mechanism of WISP1 involvement in HNC. CCN, centralized coordination network; CTGF, connective tissue growth factor; HNC, head and neck cancer; WISP1, Wnt‐1 induced secreted protein‐1.

In summary, despite the limited research on the CCN protein family in HNC, existing studies indicate that CCN2 and CCN4‐6 actively participate in the proliferation, invasion, migration, and apoptosis of HNC cells. Moreover, their biological functions are mediated through interactions with diverse signaling cascades or non‐coding RNAs. Given the distinctive four‐module organization structure of the CCN protein family and their independent and/or complementary roles, it is reasonable to hypothesize that other understudied CCN proteins also play a pivotal role in the initiation and progression of HNC. The mechanism of CCN protein family involved in HNC is shown in Figure 2.

## 5 THROMBOSPONDINS

THBS, initially discovered in platelets, constitute a family of oligomeric, multi‐domain calcium‐binding glycoproteins that possess the ability to interact with other ECM components and cell surface receptors for their functional roles.^74^ This protein class actively promotes wound healing, angiogenesis, connective tissue organization, and synaptogenesis.^13^ THBS encompasses five family members (THBS‐1‐5) that share conserved characteristic domains and assemble into trimeric and pentameric oligomer structures in a modular manner.^75^ The recognized domains of THBS include the amino terminus, vWc domain, thrombospondin repeats (type I repeats), epidermal growth factor‐like repeats (type II repeats), calcium‐binding domains (type III repeats), and the carboxyl terminus region.^76^ The five members of THBS exhibit shared type II and type III repeats, whereas THBS‐1/2 possess three type I repeats and form trimers due to the presence of the vWc domain. Conversely, THBS‐3‐5 lack type I repeats characteristic of pentameric proteins.^77^ Investigations on THBS in HNSCC have demonstrated that THBS‐1 exhibits high expression levels in HNC cells and is associated with cancer invasiveness and angiogenesis. However, no comprehensive description has been provided for THBS‐2‐5 thus far.

In a study investigating Gene Ontology enrichment profiling and Ingenuity Pathway Analysis, Jerhammar et al.^78^ identified 14 hub genes, including FN1, SERPINE1, and THBS‐1, as potential biomarkers of radiosensitivity in HNSCC. Among these markers, THBS‐1 exhibited the highest score. Similarly, Sepiashvili et al. conducted mass spectrometry‐based proteomic analysis of conditioned medium from HNC cell lines followed by RT‐PCR, Western Blot, immunohistochemistry, and ELISA to validate the selected markers.^79^ Their findings revealed that elevated tumor expression levels of five markers including THBS‐1 were significantly associated with poor disease‐free survival and an increased risk of disease progression or recurrence. In addition, Shrivastava et al. successfully isolated cancer stem cell‐like cells (oralspheres) from HNC cells and observed a significantly enhanced tumor growth in oralspheres compared to parent cells. Subsequently, they conducted an analysis of the molecular determinants associated with oralspheres. Notably, there were substantial differences in the expression levels of angiogenesis and invasive marker genes such as angiopoietin1, integrinβ3, MMP9, and THBS‐1.^80^ These findings provide compelling evidence for a close association between THBS‐1 and the occurrence, progression, and prognosis of HNC. Furthermore, Albo et al., in their investigation on the invasiveness of HNC cells,^81^ discovered that THBS‐1 could up‐regulate urokinase plasminogen activator receptor (uPAR) by three‐fold and urokinase plasminogen activator (uPA) by four‐fold. Importantly, both uPA and uPAR have been independently validated as prognostic indicators for invasive tumor behavior.^82^ THBS‐1 up‐regulated the invasiveness of HNC cells, but down‐regulated the invasiveness of tumor cells mediated by THBS‐1 when uPAR was inhibited.

Although the impact of THBS‐2‐5 on HNC remains unexplored, it is worth noting that THBS‐1 has been extensively linked to cancer invasiveness and angiogenesis. Consequently, investigating the potential role of THBS‐1 in this context presents a promising avenue for opportunistic research, enabling a deeper understanding of the intricate involvement of THBS proteins in HNC. Moreover, such investigations hold significant potential for identifying reliable molecular targets that can enhance both diagnosis and prognosis strategies for HNC patients.

## 6 SPARC

**FIGURE 3:**
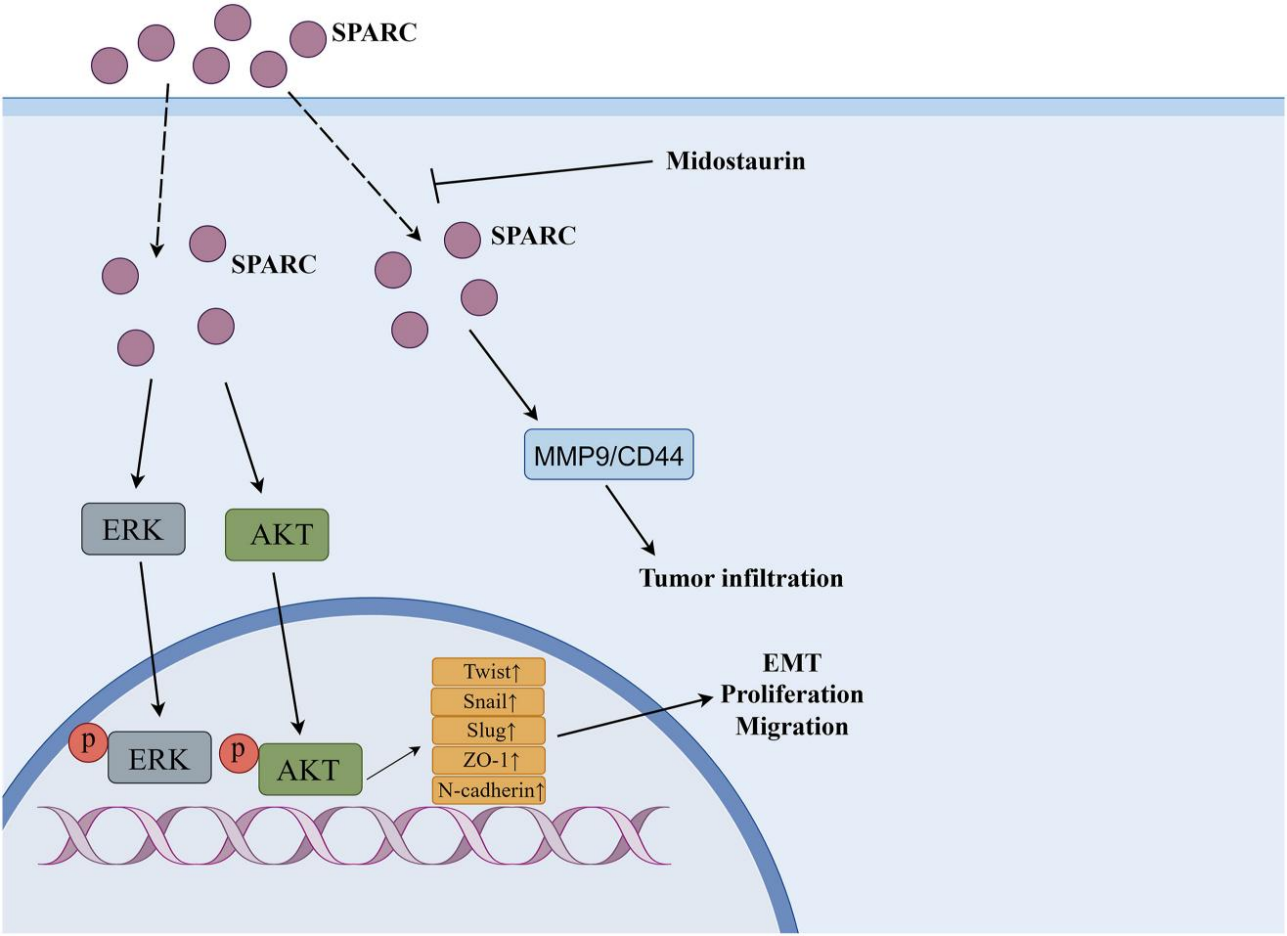
Mechanism of SPARC protein family involved in HNC. HNC, head and neck cancer; SPARC, secreted proteins acid and rich in cysteine family.

Like THBS, SPARC is among the earliest proteins identified as MCPs. The SPARC protein family encompasses eight members: SPARC, SPARCL1, SMOC‐1‐2, SPOCK‐1‐3, and FSTL1. All of these proteins possess a follicle‐like domain and an extracellular calcium binding domain.^83^ Members of the SPARC protein family exert regulatory control over ECM assembly and deposition, disrupt cell adhesion, impede cell proliferation, modulate the activity of extracellular proteases, regulate multiple signaling pathways, and are implicated in various human diseases including cancer and autoimmune disorders.^84^, ^85^ Notably observed in normal tissues undergoing development or remodeling processes as well as tissue repair mechanisms; however, in numerous cancerous conditions there is up‐regulation of SPARC expression which significantly contributes to tumor progression.^86^, ^87^ It is noteworthy that a substantial body of research has consistently demonstrated the involvement and significant role of SPARC in the pathogenesis and progression of HNC. Chin et al. conducted a comprehensive analysis, comparing the expression profiles of over 13,000 unique genes in seven matched HNC cells with normal oral mucosal cells. Their meticulous cDNA microarray analysis revealed a consistent upregulation of SPARC during the transition from normal mucosa to tumor tissue, which was further validated through immunohistochemistry. Furthermore, their rigorous univariate and multivariate survival analyses identified SPARC as a robust independent prognostic marker for patients with HNC.^88^ The investigation of Chin serves as a pioneering example in identifying SPARC as an autonomous prognostic marker for HNSCC. Chang et al. discovered that the expression of SPARC was higher in grade IV tumor samples compared to I‐III grade HNC samples. Following SPARC treatment, noticeable proliferation and migration were observed in HNC cells, accompanied by the induction of EMT through activation of the AKT signaling pathway by SPARC.^89^ Furthermore, there is evidence linking SPARC to targeted therapy for HNC. Desai and colleagues demonstrated that^90^ patients with positive SPARC expression exhibited a greater response to nab‐paclitaxel than those who were negative for SPARC. Although this study is preliminary, the data support the notion that overexpression of SPARC may be associated with a favorable response to nab‐paclitaxel treatment. In a recent study, Lin et al. conducted an extensive analysis of various sequencing databases, including microarray and single‐cell RNA sequencing database, to identify SPARC/MMP9/CD44 as a targeted genetic feature of HNSCC. The authors observed significantly higher expression levels of SPARC/MMP9/CD44 features in HNC compared to adjacent normal tissues. Furthermore, employing genomic methods, they identified midostaurin as a promising drug candidate that targets SPARC/MMP9/CD44, thereby highlighting the potential therapeutic value of targeting SPARC in HNC.^91^ The mechanism of SPARC protein family involved in HNC is shown in Figure 3.

## 7 PERIOSTIN

Vitamin K serves as an anti‐hemorrhagic factor and plays a crucial role in the regulation of numerous diseases. Its primary mechanism involves post‐translational modification of multiple glutamate residues to γ‐carboxyglutamate (Gla) residues within various proteins, enabling them to function effectively. These proteins are classified as vitamin K‐dependent or members of the Gla protein family.^92^ Within this family, there exist 17 proteins, including notable ones such as Periostin, matrix Gla protein (MGP), osteocalcin/bone Gla protein, and prothrombin. While the Gla protein family is primarily recognized for its significance in bone metabolism and calcification, recent studies have highlighted the vital roles played by Periostin and MGP in ECM metabolism, inflammation, fibrosis, allergic diseases, and cancer.^14^, ^93^ In the context of HNC, Periostin emerges as the most frequently implicated member, while the understanding of other Gla protein family members' roles in HNC remains limited. Periostin typically exhibits low expression levels in normal tissues and cells, but demonstrates high expression at pathological sites. Initially identified as osteoblast specific factor‐2, Periostin was isolated from mouse preosteoblast cell line MC3T3‐E1 through subtraction from fibroblast cell line NIH3T3.^94^, ^95^ It encompasses an N‐terminal secretory signal peptide, an EMI domain, a tandem repeat sequence comprising four FAS1 domains, and a hydrophilic carboxyl terminal domain. These distinct domains have been experimentally confirmed to interact with various proteins and exert diverse biological functions.^96^, ^97^ Although Periostin does not directly participate in ECM formation, its presence within this intricate structure is crucial for mediating cellular interactions with the surrounding microenvironment. In terms of disease progression, Periostin exerts its biological function by modulating various signaling pathways through integrin binding, thereby either stimulating or inhibiting their activation.^14^, ^93^

By comparing the gene expression profiles between parental HNC cells and highly invasive clones, Kudo et al. identified Periostin as a crucial factor promoting invasion in HNC. They observed that overexpression of Periostin significantly enhanced the invasiveness and autonomous growth of HNC cells both in vitro and in vivo.^98^ Moreover, utilizing an in situ mouse model of HNC, they demonstrated that Periostin‐overexpressing cells exhibited spontaneous metastasis to cervical lymph nodes and lungs due to their heightened invasiveness. Notably, elevated expression levels of Periostin were detected in HNC tissues compared to normal tissues. Subsequently, another study by the same group^99^ revealed that upregulation of Periostin led to increased mRNA expression of vascular endothelial growth factor‐C (VEGF‐C) within HNC cells, which is known as a pivotal activator for tumor lymphangiogenesis during the process of lymphatic metastasis.^100^ Kudo et al. demonstrated the induction of tube formation in lymphoendothelial cells through the use of conditioned media derived from Periostin‐overexpressing cells. Their findings revealed that Periostin overexpression significantly promoted tube formation, thereby implicating its role in facilitating lymphatic metastasis of HNC. Similarly, Deraz et al.^101^ reported that ectopic upregulation of MMP‐10 enhanced invasion in HNC cells, whereas knockdown of MMP‐10 inhibited invasion. Notably, microarray analysis identified MMP‐10 as a commonly upregulated gene following Periostin overexpression in HNC cells. Furthermore, the observed inhibition of HNC cell invasion upon knockdown of MMP‐10 further underscores the crucial involvement of Periostin in the initiation and progression of HNC. Qin and colleagues discovered that Periostin exhibits a high concentration in the ECM of cancerous tissue, predominantly originating from cancer‐associated fibroblasts.^102^ Their findings demonstrate a significant upregulation of Periostin in HNC, particularly in tissues with lymph node metastasis, aligning with previous studies.^99^ Furthermore, their research highlights the direct stimulation of cancer cell growth, migration, and invasion through TGF‐β3‐induced upregulation of Periostin expression. Notably, Periostin's involvement in HNC is mediated by diverse signaling pathways to execute its biological functions. Yu and his colleagues discovered that^103^ Periostin, secreted by cancer‐associated fibroblasts, binds to PTK7 on the cell membrane, leading to phosphorylation of GSK‐3β and hypophosphorylation of β‐catenin through LRP6. Consequently, there is an accumulation of β‐catenin and its translocation into the nucleus, thereby promoting metastasis in squamous cells of HNC. Liu et al. also reported a significant correlation between Periostin expression levels with pathological grade and lymphatic metastasis in HNC. Inhibition of Periostin expression resulted in decreased levels of p‐PI3K, p‐Akt, and p‐mTOR proteins suggesting that Periostin mediates the PI3K/Akt/mTOR signaling pathway involved in promoting proliferation, invasion, survival, tumorigenicity as well as migration in HNC cells.^104^ Furthermore, it has been documented that Periostin regulates EMT in HNC via mediation of the ERK/Akt signaling pathway.^105^

**FIGURE 4:**
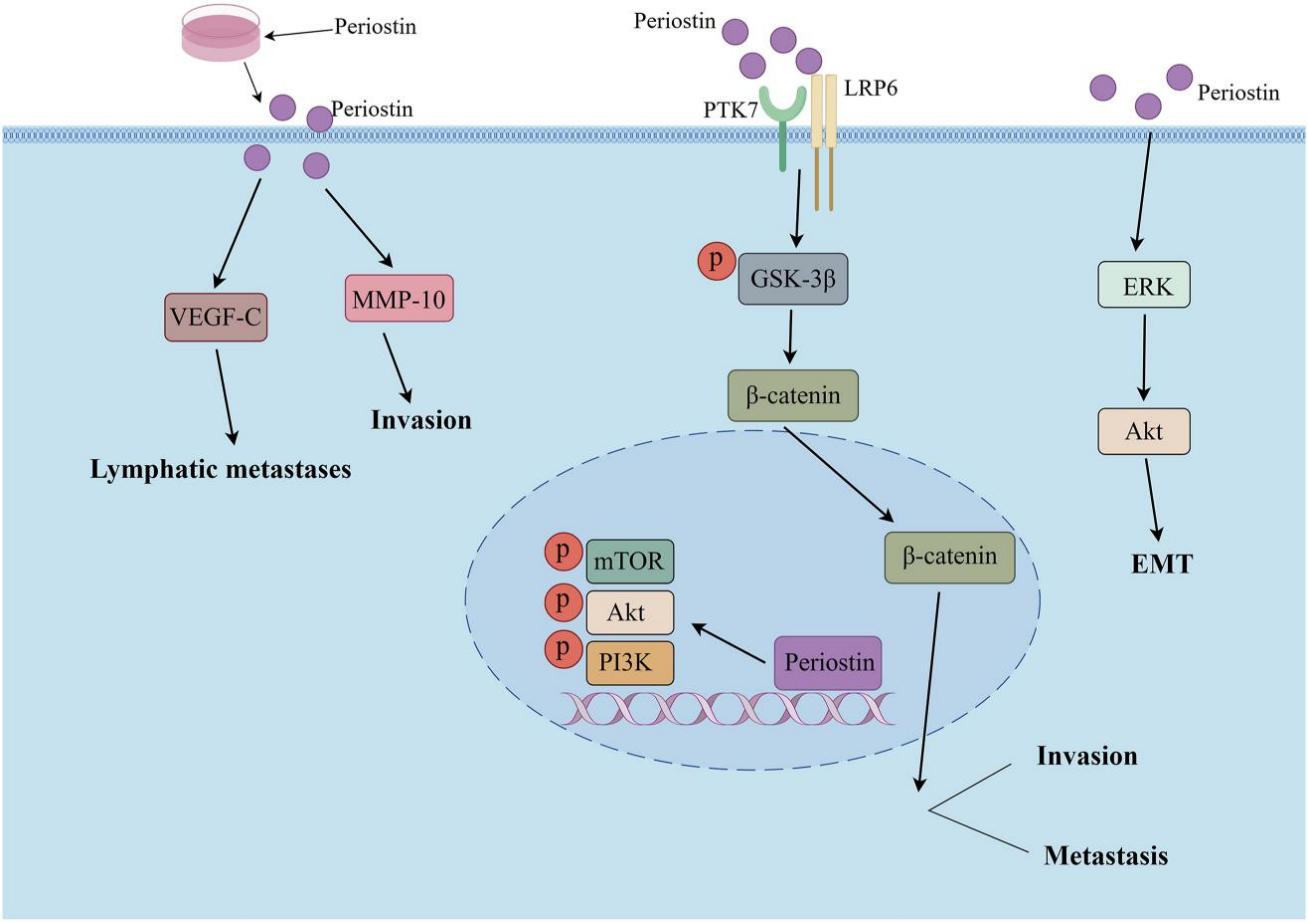
Mechanism of Periostin involved in HNC. HNC, head and neck cancer.

In conclusion, Periostin plays a pivotal role in the pathogenesis of HNC. It not only facilitates the proliferation, migration, invasion, and EMT of HNC cells but also promotes lymphatic metastasis. Moreover, Periostin exhibits close associations with various signaling pathways within HNC cells, thereby presenting itself as a promising target for prognosis and therapeutic interventions. Regrettably, there is currently no available literature on other members of the Gla protein family in relation to HNC; hence further investigation into this area is warranted. The mechanism of Periostin involved in HNC is shown in Figure 4.

## 8 OSTEOPONTIN

Osteopontin, the prominent protein within the SIBLING protein family, is accompanied by four other members: BSP, DSPP, DMP1, and MEPE. This particular protein family predominantly resides in bone and dentin tissues while playing a pivotal role in osteogenesis and dentinogenesis.^106^ All SIBLING proteins encompass an Arg‐Gly‐Asp motif that facilitates binding to integrin αvβ3—an essential aspect of their hydrophilic structure and cellular organization.^107^ Interestingly enough, unlike conventional protein families, the SIBLING protein family exhibits minimal inherent sequence homology. Furthermore, their distribution and functionality heavily rely on post‐translational modifications such as phosphorylation and glycosylation.^106^, ^108^ Among the SIBLING protein family, Osteopontin has emerged as the most extensively investigated protein, thus research on this protein family in the context of HNC has predominantly focused on Osteopontin. Osteopontin, also known as secreted phosphoprotein 1, is secreted by various cell types including osteoclasts, chondrocytes, synoviocytes, macrophages, lymphocytes, epithelial cells and vascular smooth muscle cells. It is present in mineralized tissues and ECM within bodily fluids.^109^, ^110^ Numerous studies have reported the involvement of Osteopontin in diverse pathological conditions such as myocardial infarction, atherosclerosis, kidney damage and diabetes. Additionally, it has been implicated in chronic inflammatory diseases like osteoarthritis and lumbar disc degeneration.^111^ Additionally, a plethora of literature reports have established a strong correlation between Osteopontin and the development as well as progression of various cancer types. Remarkably, in the majority of malignancies, heightened expression levels of Osteopontin or its isoforms frequently serve as an ominous indicator for unfavorable prognosis.^112^

Eto and colleagues were the pioneers in quantitatively measuring serum Osteopontin levels in patients with HNC using a sandwich enzyme immunoassay. Their findings revealed a significantly higher average Osteopontin level (99.5 ng/mL) in HNC patients compared to the control group (55.3 ng/mL),^113^ particularly among those at advanced clinical stages (stage III or IV). Interestingly, they also observed that Osteopontin expression levels did not correlate with serum squamous cell carcinoma antigen levels. Additionally, Bie et al. corroborated the expression of Osteopontin in HNC patients. In vivo xenograft experiments were conducted to assess the expression of Osteopontin in HNSCC tissues using immunohistochemistry and immunofluorescence. The findings revealed a significant upregulation of Osteopontin in HNC tissues, which was further confirmed by Western Blot analysis demonstrating higher levels of Osteopontin expression compared to adjacent normal tissues.^114^ Moreover, the authors postulated that elevated levels of Osteopontin as indicated by KM curve analysis are associated with unfavorable survival outcomes among patients. In another experiment aimed at investigating the potential correlation between Osteopontin levels and the status of cervical lymph node metastasis in HNC patients with different primary sites, Maleš et al. conducted a measurement of plasma Osteopontin levels. The results revealed a significant elevation in Osteopontin levels within the plasma of patients with positive lymph nodes compared to those with negative cervical lymph nodes.^115^ Consequently, they proposed that Osteopontin might play a crucial role in the development of cervical lymph node metastasis. Furthermore, several studies have demonstrated that Osteopontin also contributes to enhancing the prognosis of HNC patients undergoing chemotherapy and radiotherapy treatments. Snitcovsky et al. employed an ELISA kit to quantify plasma Osteopontin levels in patients diagnosed with HNSCC who underwent platinum‐based chemoradiotherapy prior to and post‐treatment completion. The authors observed that individuals with advanced T and N stages exhibited elevated pre‐treatment plasma Osteopontin levels compared to those at early‐stage disease. However, intriguingly, they discovered no significant alteration in Osteopontin levels within a limited cohort of patients assessed before and after chemoradiotherapy.^116^ Snitcovsky et al. proposed the possibility that transient tumor destruction might induce a surge in plasma Osteopontin levels, released by dying cells or the tumor stroma. Another potential explanation is that some patients may have residual disease but still achieve a complete response according to conventional assessment methods. Polat et al. investigated the impact of perioperative changes in Osteopontin plasma levels on the prognosis of radiotherapy for HNC. Their findings revealed that^117^ HNC patients exhibited elevated Osteopontin plasma levels immediately after surgery, which subsequently decreased to preoperative levels 4 weeks later when adjuvant radiation therapy typically commences. Given Osteopontin's prognostic value for malignant behavior and its influence on radiation response, this could account for the prognostic effects of preradiation Osteopontin in both primary and postoperative treatment settings. Furthermore, previous studies have linked Osteopontin with tumor hypoxia and malignant phenotypes. Bache et al. demonstrated a significant association between positive Osteopontin staining and low hemoglobin levels, high expression of HIF‐1α, and elevated serum VEGF in advanced HNC using immunohistochemistry.^118^ Furthermore, when combining the immune groups of Osteopontin, HIF‐1α, and CAIX into hypoxia curves, a robust and statistically significant impact on overall survival was observed. Coincidentally, Wohlleben et al. also reported strong regulation of osteopontin by hypoxia and to a lesser extent by radiation in HNC.^119^ These findings collectively indicate that Osteopontin is highly expressed in HNC tissues and closely associated with lymphatic metastasis and prognosis. Additionally, numerous literature reports suggest that Osteopontin can influence the progression of HNC through its interactions with multiple signaling pathways.

Chien et al. discovered a positive correlation between Osteopontin and Aurora‐A in HNSCC through an analysis of microarray profiles available in the database.^120^ Aurora‐A, also known as STK15 or STK6, is frequently amplified or overexpressed in various human cancers, including HNC.^121^, ^122^ The authors observed that stimulation of HNC cells with Osteopontin resulted in increased expression of Aurora‐A localized at the centrosome. Furthermore, they found that this upregulation of Aurora‐A induced by Osteopontin stimulation enhanced the invasive potential of HNC cells by augmenting ERK1/2 activity. Simultaneously, it was also observed that Osteopontin can induce the activation of ERK1/2, while this effect can be inhibited by anti‐CD44. Subsequently, Chien successfully established a significant positive correlation between Osteopontin‐Aurora‐A and ERK1/2 through immunohistochemistry and Western Blot analysis conducted on human invasive HNC specimens. Consequently, these findings not only suggest that Aurora‐A serves as a target within the Osteopontin/CD44/ERK1/2 pathway but also emphasize its significance as an essential prognostic factor in HNC. Qin et al. employed a co‐culture methodology to ascertain that cancer‐associated fibroblasts secrete IL‐6, which serves as the principal upstream molecule responsible for triggering tumorous Osteopontin. The interaction between fibroblasts and cancer cells induces an upregulation of neoplastic Osteopontin expression through IL‐6 stimulation, thereby facilitating the growth, migration, and invasion of cancer cells.^123^ Significantly, they also discovered that in HNC cells, tumorous Osteopontin directly enhances the activity of the NF‐κB signaling pathway by binding to integrin αvβ3 while indirectly modulating it through upregulation of MMPs, uPA, and ICAM‐1 expression in cancer cells. This creates a tumor‐promoting microenvironment conducive to HNC progression. Consequently, the combination of IL‐6 and Osteopontin holds promise as both a prognostic and diagnostic indicator as well as a potential therapeutic target for patients with HNC. Furthermore, investigations conducted by Liu et al. demonstrated that the expression level of RUNX1 escalates in patients with HNC as the disease progresses.^124^ RUNX1 is widely acknowledged as a crucial transcription factor associated with cellular developmental processes, stem cell biology, and tumorigenesis.^125^ Profiling gene expression in various cancers including metastatic lung, breast, prostate, colorectal, uterine, and ovarian adenocarcinomas has indicated that patterns of RUNX1 expression might serve as predictive markers for metastasis.^126^ Remarkably, their findings revealed that silencing of RUNX1 significantly impeded the malignant progression of HNC cells both in vitro and reduced Osteopontin expression while also diminishing the tumorigenicity of HNC cells. Furthermore, the study also demonstrated, through luciferase reporter experiments and chromatin immunoprecipitation assays, that RUNX1 exhibits direct binding to the promoter region of Osteopontin. This interaction subsequently activates the MAPK signaling pathway, thereby contributing to disease progression and metastasis. Additionally, their previous investigation revealed that^127^ lentivirus‐mediated overexpression of Osteopontin upregulates KRAS/MEK pathway activity, consequently impacting malignant progression, prognosis, and cetuximab resistance in HNC.

**FIGURE 5:**
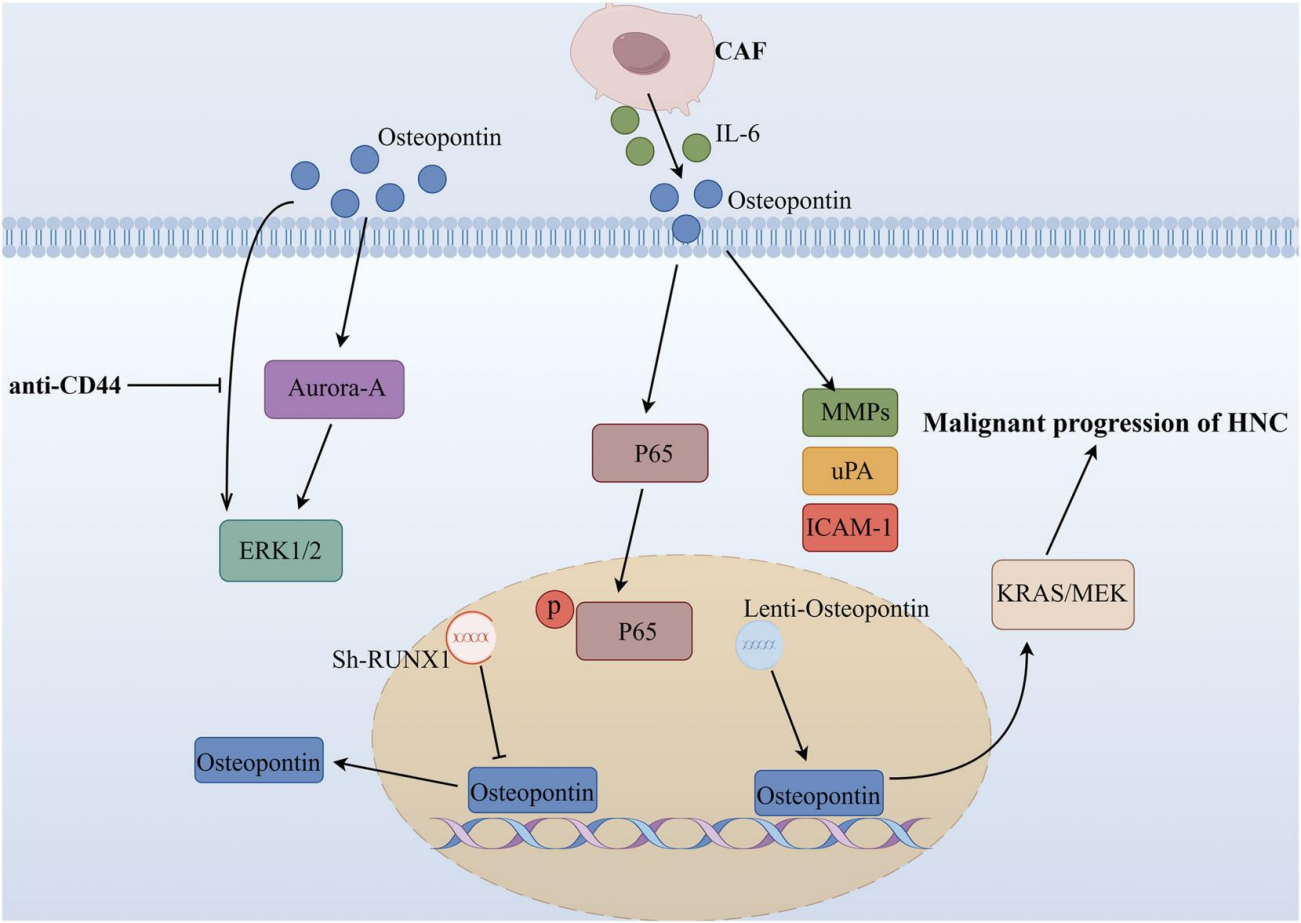
Mechanism of Osteopontin involved in HNC. HNC, head and neck cancer.

In conclusion, based on the existing research findings, Osteopontin emerges as a pivotal target influencing the onset and progression of HNC. This protein not only exhibits high expression levels in HNC cells but also demonstrates a close association with cervical lymph node metastasis, thereby serving as an indicator for predicting the prognosis of patients undergoing radiotherapy and chemotherapy. Furthermore, Osteopontin is intricately linked to tumor hypoxia. At the molecular level, it acts as both an upstream and downstream regulator of certain molecules involved in the molecular mechanisms underlying HNC. Additionally, it modulates various signaling pathways including ERK1/2, NF‐κB, and KRAS/MEK pathways to impact the malignant progression and prognosis of HNC. Regrettably, there is currently no available literature reporting on other members belonging to the SIBLING protein family in relation to HNC, thus warranting further investigation in this area. The mechanism of Osteopontin involved in HNC is shown in Figure 5.

## 9 OTHER MCPS

Tenascin is a multifunctional MCP. Its conserved structure includes N‐terminal seven repeat sequences, EGF‐like domains and a series of fibronectin type III domains, which are important for the interaction between cell surface proteins and soluble factors in ECM.^128^ Tenascin has been shown to mediate cell adhesion, migration, matrix assembly, up‐regulation of fibrogenic cytokines and myofibroblast differentiation.^129^ Tenascin has four members (tenascin‐C, ‐R, ‐W and‐X) in vertebrates.^130^ But in the research of HNC, Tenascin‐C (TNC) is the focus of the research. In the earliest study, Pauli et al.^131^ evaluated serum TNC level in 92 patients with primary HNC and 28 patients with recurrent TNC. When they measured TNC levels using ELISA, they found that serum TNC levels were significantly higher in patients with higher tumor stages or recurrent diseases than in healthy controls. Yang et al. studied the immunohistochemical expression of TNC in 136 esophageal squamous cell carcinoma tissue samples. They found that TNC may be a reliable and important prognostic factor in esophageal squamous cell carcinoma.^132^ Routray and colleagues used bioinformatics to predict candidate genes, and combined with proteomics and immunohistochemistry to verify their existence and participation in the pathway of oral squamous cell carcinoma that predicted invasion and metastasis, they identified TNC as an important gene for metastasis and invasion.^133^ It was also reported that silencing the expression of TNC down‐regulated the expression of cancer stem cell‐like cell marker SOX2 and EMT‐related marker Snail, while Perifosine, an inhibitor of Akt signal, could inhibit the protein expression of HIFA α and TNC in esophageal squamous cell carcinoma cells.^134^ This suggests that TNC may enhance the characteristics of cancer stem and promote EMT‐like changes through the Akt/HIF1 α axis. In addition, Spenlé et al. reported that TNC coordinates immunosuppressive tumor microenvironment in oral squamous cell carcinoma.^135^ In this study, through TLR4, TNC increased the expression of CCR7 in ^CD11c+^myeloid cells. CCL21 in lymphoendothelial cells was induced by integrin α9β1 and bound to CCL21, and TNC fixed ^CD11c+^cells in the matrix. Ablation of TNC or CCR7 blockade can inhibit the characteristics of lymphoid immunosuppressive matrix and reduce tumor growth, progression and metastasis.

Fibulin family proteins share C‐terminal fibrin module and calcium‐binding EGF‐like domain. Eight members of the family have been identified in mammals: Fibulin‐1, ‐2, ‐3, ‐4, ‐5, ‐7, and Hemicentin‐1 and‐2.^136^ Fibulin‐3 is encoded by the EFEMP1 gene and has been shown to be closely related to a variety of cancers.^137^, ^138^ Guo et al. found that Tiam1 transfection stimulates the expression of MMP‐7 by accelerating the nuclear translocation of β‐catenin in oral squamous cell carcinoma cell line CAL27, which is contrary to the function of Fibulin‐3. Tiam1 has been reported as a potential oncogene in oral squamous cell carcinoma. In this study, Tiam1 directly interacts with Fibulin‐3 to compete for the nuclear translocation of β‐catenin, and then stimulates the expression of MMP‐7 through the interaction of TCF‐4 domain to promote EMT.^139^ In addition, Chen et al.^140^ found that the expression of Fibulin‐4 is increased in esophageal squamous cell carcinoma, and its down‐regulation inhibits the proliferation, invasion and migration of cancer cells in vitro and in vivo. Inhibition of Fibulin‐4 expression inhibits cell protective autophagy by activating Akt‐mTOR signaling pathway, thus enhancing cell sensitivity to apatinib.

Galectins, encoded by the LGALS gene, are a group of low‐molecular‐weight β‐galactoside‐binding proteins that belong to the endogenous lectin family.^141^, ^142^ They possess a conserved amino acid sequence and carbohydrate recognition domain.^143^ Currently, 16 members of the Galectins family have been identified. These proteins play crucial roles in various biological processes including cell cycle regulation, immunity modulation, proliferation control, differentiation induction, pre‐mRNA splicing regulation, cell‐cell and cell‐matrix adhesion facilitation as well as apoptosis modulation. Moreover, they also participate in signal transduction regulation^44^ and significantly contribute to cancer progression and metastasis through mediating interactions between tumors and the tumor microenvironment.^144^ Chawla et al.^145^ reported a negative correlation between the percentage of Galectin‐1 expression in tumor areas with lymphocyte infiltration and survival/recurrence rates. Similarly, Saussez and colleagues^146^ observed an up‐regulation of Galectin‐3 during the progression of pharyngeal and laryngeal squamous cell carcinoma. Furthermore, Coppock et al. conducted a tissue microarray analysis to evaluate Galectin‐3 expression in high‐risk HPV (+) and HPV (−) HNC tissues as well as regional lymph node metastases. They discovered that compared to HPV (−) HNC tissues, there was higher expression of Galectin‐3 in HPV (+) HNC tissues, suggesting its potential as a therapeutic target for HPV (+) HNC treatment. However, no significant difference in Galectin‐3 expression was observed between primary tumors and regional lymph node metastases.^147^ In addition to Galectin‐1 and Galectin‐3, Chen et al. discovered a positive correlation between the expression of Galectin‐7 and the stage of HNSCC using immunohistochemistry. Furthermore, they observed that HNC patients with positive nuclear staining for Galectin‐7 had significantly poorer survival rates compared to those with positive cytoplasmic staining. Additionally, their study revealed that Galectin‐7 interacts with the HSP40 co‐chaperone protein Tid1, thereby promoting Tid1‐mediated ubiquitination and proteasomal degradation of Galectin‐7. Moreover, it was found that Galectin‐7 enhances the transcriptional activity of the transcription factor TCF3 by upregulating MMP‐9 expression, thus playing a pivotal role in facilitating tumorigenesis and metastasis progression.^148^

Laminin, a group of multi‐domain, non‐collagenous ECM glycoproteins found in the basement membrane, encompasses 16 distinct isoforms that have been identified thus far. These isoforms play crucial roles in regulating various cellular processes such as adhesion, differentiation, migration, phenotype maintenance, self‐renewal, tumor growth and metastasis. Additionally, they exhibit potential resistance to apoptosis through both integrin and non‐integrin signaling receptors.^128^, ^149^ Among these laminins, Laminin‐332 holds significant importance in maintaining normal epithelial homeostasis; however, it is frequently overexpressed by malignant cells and has been associated with increased tumor aggressiveness and unfavorable prognosis. Its involvement promotes cancer cell survival along with facilitating EMT, motility, tissue invasion and migration—all contributing to the progression of cancer.^150^ In the investigation of HNC, Kinoshita et al. discovered an up‐regulation of Laminin‐332 in tumor tissues, while miRNA‐218 exhibited its ability to inhibit tumor cell migration and invasion by specifically targeting Laminin‐332.^151^ Furthermore, their subsequent study demonstrated that miRNA‐29 effectively targets the Laminin‐332‐integrin α6β4 signaling pathway to suppress cancer cell migration and invasion.^152^ Previous research has also elucidated that the interaction between Laminin‐332 and integrin α6β4 triggers multiple signaling cascades within tumor cells, promoting both cell migration and cancer cell survival.^153^ Additionally, it has been reported that under low fluid shear force conditions (0.07 dyn/cm^2^), HNC squamous cells can bind with laminin and engage in interactions mediated by β1 integrins (including α2β1, α3β1, and α6β1) to regulate intracellular calcium signaling transduction.^154^ Therefore, a comprehensive understanding of the regulatory mechanisms underlying tumor suppressive miRNAs and their impact on Laminin‐332‐integrin signaling is crucial for elucidating the invasive and progressive nature of HNC, ultimately paving the way for more efficacious therapeutic interventions in this disease.

The involvement of the uPA‐plasminogen system in the progression of various cancers has been extensively documented.^155^ Plasminogen activator inhibitor‐1 (PAI‐1) exerts its inhibitory effect on uPA by directly binding to it. Additionally, PAI‐1 plays a crucial role in safeguarding matrix metalloproteinases (MMPs) against plasmin‐mediated activation and protecting ECM proteins from proteolytic degradation.^156^, ^157^ Nuszkiewicz et al. propose that PAI‐1 may be implicated in the occurrence and development of HNC.^158^ Furthermore, Hakelius et al., through their research, have observed an upregulation of PAI‐1 expression in HNC cells, which can be downregulated by IL‐1α stimulation.^159^ In addition, Lee and colleagues employed the PAI‐1 specific inhibitor Tiplaxtinin and lentiviral transfection to effectively suppress the expression of PAI‐1. Their findings revealed that down‐regulation of PAI‐1 expression can effectively inhibit the expression of SOX2, thereby impeding the self‐renewal ability of tumor initiation cells in HNC lines.^160^ These results suggest that PAI‐1 could potentially serve as a targeted regulatory factor for tumor‐initiating cells in HNC, thus highlighting the therapeutic potential of inhibiting PAI‐1 as a strategy to target these cells. Furthermore, another study demonstrated that three human HNC cell lines (BHY, CAL27, FaDu) exposed to hypoxia (<0.5% O_2_) for 72 h exhibited a subsequent reoxygenation under normoxic conditions for 24 h. Interestingly, this reoxygenation led to a significant upregulation in cellular secretion of PAI‐1 and formation of uPA‐PAI‐1 complex.^161^

## 10 DISCUSSION AND CONCLUSION

HNSCC primarily originates from the mucosa lining the upper respiratory tract, including regions such as lips, oral cavity, salivary glands, larynx, nasopharynx, hypopharynx, and oropharynx. It is an invasive malignant tumor associated with significantly high morbidity and mortality rates. Risk factors for HNSCC encompass smoking habits, alcohol consumption patterns, exposure to environmental pollutants along with viral infections like HPV and EB viruses. Currently available treatment modalities for HNC comprise surgical interventions in combination with radiotherapy or chemotherapy; however, long‐term survival rates remain considerably low among patients. Notably, only two molecular‐based treatments namely cetuximab and monoclonal anti‐PD‐L1 have gained approval for treating HNC at present. Therefore, it is imperative to identify molecular biomarkers capable of predicting the progression of HNSCC in order to enhance survival rates and discover novel therapeutic targets. MCPs exhibit potent biological functionality, with an increasing number of studies highlighting their involvement in the pathogenesis and advancement of various cancer types, particularly HNC. Consequently, this review aims to elucidate the role of MCPs and provide an update on the research progress pertaining to the MCP family in HNC, with a focus on identifying new biomarkers for this disease. Primarily through binding to matrix proteins, cell surface receptors or other molecules, MCPs predominantly activate specific proteins and molecules while modulating diverse signaling pathways that ultimately influence disease initiation and progression. MCPs are primarily categorized into seven families: THBS, fibulins, SPARC family, CCN family, TNs, SIBLING family, and the Gla protein family. Notably, pivotal molecules such as CCN2, THBS‐1, SPARC, POSTN and Osteopontin have been implicated in the pathogenesis and progression of HNSCC. By virtue of their distinctive structure and functionality, they facilitate tumor proliferation, invasion dynamics including EMT induction and lymphatic metastasis in HNC cells. In addition, they can also bind to integrins and modulate various signaling pathways to fulfill their biological functions.

Moreover, MCPs can serve as targets not only for other molecules influencing the progression of HNC but also as independent prognostic indicators for HNC. Although the current research on MCPs has not appeared in preclinical or clinical studies, based on the existing basic research, we can also determine that MCPs can be used as a prognostic indicator and target of HNC to a large extent. In future research, we can pay more attention to these MCPs themselves or the molecules and drugs that can target them. For example, as a target of miR‐384, miR‐384 can directly target WISP1 to regulate the proliferation and apoptosis of HNC cells.^69^ As the top targeted drug of SPARC/MMP9/CD44,^91^ Midostaurin can be regarded as the focus of preclinical research. In addition, Aurora‐A can also be used as an important target to further study the biological function of Osteopontin/CD44/ERK1/2.^120^ Although numerous MCPs have yet to be explored in the context of HNC research, based on the concise emphasis provided in this review, MCPs hold great promise for the future of HNC. Ultimately, we anticipate that further investigations will yield a more comprehensive and profound understanding of the role played by MCPs in the molecular mechanisms underlying HNC, thereby facilitating the development of valuable diagnostic or therapeutic agents aimed at enhancing individuals' quality of life.

## AUTHOR CONTRIBUTIONS

**Yunsheng Wang** and **Jincai Xue**: Conceptualization. **Yunsheng Wang** and **Xudong Liu**: Data curation. **Xingyue Wang** and **Jiyong Lu**: Writing – original draft preparation. **Youxin Tian** and **Qinjiang Liu**: Writing – review and editing. **Jincai Xue**: Supervision. **Yunsheng Wang** and **Xudong Liu** had equal contributions to the article. All authors have read and agreed to the published version of the manuscript.

## CONFLICT OF INTEREST STATEMENT

The author declares no conflict of interest.

## INSTITUTIONAL REVIEW BOARD STATEMENT

Not applicable.

## ETHICS STATEMENT

This article does not involve any ethics review.

## INFORMED CONSENT STATEMENT

Not applicable.

## Data Availability

Data sharing is not applicable to this article as no new data were created or analyzed in this study.
